# Cerebral Circulation Time Is a Potential Predictor of Disabling Ischemic Cerebrovascular Events in Patients With Non-disabling Middle Cerebral Artery Stenosis

**DOI:** 10.3389/fneur.2021.653752

**Published:** 2021-05-07

**Authors:** Zhenze Chen, Mingchun Li, Zhihuan Wu, Min Zhang, Guomei Weng, Minzi Li, Rongxin Liao, Peng Zhao, Jianming Wu, Shuzhen Zhu, Qing Wang, Chunguang Li, Xiaobo Wei

**Affiliations:** ^1^Department of Neurology, Zhujiang Hospital, Southern Medical University, Guangzhou, China; ^2^Department of Neurology, 1st People Hospital of Zhaoqing, Zhaoqing, China; ^3^Department of Neurology, Jiangmen Centrol Hospital, Jiangmen, China; ^4^Department of Gerontology, Integrated Hospital of Traditional Chinese Medicine, Southern Medical University, Guangzhou, China

**Keywords:** non-disabling middle cerebral artery stenosis, cerebral circulation time, digital subtraction angiography, disabling ischemic cerebrovascular events, prognosis

## Abstract

Patients with non-disabling middle cerebral artery (MCA) stenosis (ND-MCAS) are at risk for disabling ischemic cerebrovascular events (DICE) despite aggressive medical therapy. In this study, we aimed to verify whether cerebral circulation time (CCT) was a potential predictor of DICE in patients with ND-MCAS. From January 2015 to January 2020, 46 patients with ND-MCAS treated with aggressive medical therapy were enrolled for digital subtraction angiography (DSA) in this convenience sampling study. They were divided into the DICE (–) and DICE (+) groups based on the occurrence of DICE within 3 months after DSA. The CCT was defined as the time from the appearance of the MCA to the peak intensity of the Trolard vein during DSA. The rCCT (relative CCT) was defined as the ratio of the CCT of the stenotic side (sCCT) to the CCT of the healthy side (hCCT). The differences in sCCT, hCCT, and rCCT between the two groups were analyzed with Mann-Whitney U tests. Logistic regression analysis was performed to evaluate the association between the risk factors and DICE. Receiver operating characteristic (ROC) curves were constructed to assess the predictive value of rCCT in identifying DICE in ND-MCAS patients. The results showed that DICE appeared in 5 of the 46 patients within 3 months. rCCT were significantly increased in the DICE (+) group compared with the DICE (–) group [1.08 (1.05, 1.14) vs. 1.30 (1.22, 1.54), *p* < 0.001]. Logistic regression analysis found that prolonged rCCT was an independent positive prognostic factor for DICE (odds ratio = 1.273, *p* = 0.019) after adjustment for potential confounders (age, diabetes, antithrombotic use, and stenosis degree). ROC analysis showed that rCCT provided satisfactory accuracy in distinguishing the DICE (+) group from the DICE (–) group among ND-MCAS patients (area under the curve = 0.985, *p* < 0.001), with an optimal cutoff point of 1.20 (100% sensitivity, 97.6% specificity). In conclusion, prolonged rCCT is independently associated with the occurrence of DICE in ND-MCAS patients and may be used to identify individuals at risk of DICE.

## Introduction

Stenting, angioplasty and aggressive medical management are established procedures for the prevention of further ischemic events following middle cerebral artery (MCA) stenosis (1–6). In recent years, an increasing proportion of patients with non-disabling MCA stenosis (ND-MCAS) have been treated with dual antiplatelet agents (7). The evidence to support their use comes mainly from the results of the two randomized clinical trials Stenting and Aggressive Medical Management for Preventing Recurrent Stroke in Intracranial Stenosis (SAMMPRIS) and Vitesse Intracranial Stent Study for Ischemic Stroke Therapy (VISSIT), which showed that pharmacologic management is safer and has a lower incidence of ischemic events than stenting (8, 9). However, SAMMPRIS and VISSIT showed a 5.8 and 9.4% risk of stroke and death, respectively, at 30 days during the treatment with dual antiplatelet agents (2, 8, 9). Therefore, early identification and subsequent stenting or angioplasty should be carried out for patients who are at high risk of disabling ischemic cerebrovascular events (DICE) or death.

Impaired cerebrovascular reserve is an important predictor of stroke and transient ischemic attack (TIA) in patients with cerebral artery stenosis or occlusion (10–12). At present, the clinical evaluation of the cerebrovascular reserve mainly relies on acetazolamide-challenged single-photon emission computed tomography (ACZ-challenged SPECT), computed tomography perfusion imaging (CTP) and magnetic resonance (MR) perfusion-weighted imaging (PWI), which are expensive and expose the patients to radiation and contrast medium (13–15). The cerebral circulation time (CCT) derived from digital subtraction angiography (DSA) has been reported to be well-correlated with cerebrovascular reserve (12, 16–21). It can help surgeons observe the patient's cerebrovascular reserve during surgery without the need for SPECT, CTP, and PWI.

In this study, we carried out a retrospective analysis of all prospectively collected data from patients with ND-MCAS treated with aggressive medical therapy, and aimed to verify whether CCT could be used as a potential predictor of DICE for patients with ND-MCAS.

## Materials and Methods

### Patients and Study Design

The clinical and radiological data from 273 patients with MCA stenosis treated at Zhujiang Hospital of Southern Medical University, Guangzhou, China between January 2015 and January 2020 were reviewed in this convenience sampling study. After excluding 227 patients with disability or multiple vascular stenosis, we enrolled 46 patients with non-disabling unilateral MCA stenosis in the final analysis. Their demographic characteristics, stroke risk factors, clinical symptoms, medications and relevant scale scores [National Institutes of National Institutes of Health Stroke Scale (NIHSS) and Modified Rankin Scale (mRS)] were reviewed by two neurologists. According to the trial A Pooled Analysis of Clopidogrel in High-Risk Patients with Acute Non-Disabling Cerebrovascular Events (CHANCE), non-disabling ischemic stroke was defined as: 1. Transient ischemic attack (TIA); 2. Minor ischemic stroke (22, 23). Transient ischemic attack was defined as a transient episode of neurological symptoms caused by focal cerebral or retinal ischemia without radiographic evidence of acute infarction (24). Minor ischemic stroke was defined as a stroke with a NIHSS score ≤ 3 or mRS score ≤ 3 (25). Patients with ND-MCAS were allocated to the DICE (+) group if they suffered from disabling ischemic cerebrovascular events (NIHSS score > 3 or mRS score > 3) in 90 days after the onset of the stroke. Otherwise, patients were allocated to the DICE (–) group. This study was approved by the Ethics Committee of Zhujiang Hospital of Southern Medical University and conducted in accordance with the ethical standards of the 1975 Declaration of Helsinki and the 1999 National Institutes of Health Human Subjects Policies and Guidance.

### DSA Protocol and Data Analysis

All patients were enrolled for DSA with a uniform and standard protocol in Zhujiang Hospital of Southern Medical University, Guangzhou, Guangdong, China. During the DSA procedures, a 5F angio-catheter was placed in the carotid artery at the C3 vertebral body level. All of the subjects were examined by a single-plane angiographic machine (GE IGS 330, America) with a power injector (5 ml ioversol injection with 3 ml/s speed, 150 psi/kg pressure). The stenotic degree was measured according to the NASCET criteria from DSA image (26). Neuroimaging and analysis were performed independently by two trained neurologists who were blinded to the clinical conditions and were responsible for assessing all of the neuroimaging variables used in this study.

### Cerebral Circulation Time Measurement

**Figure 1:**
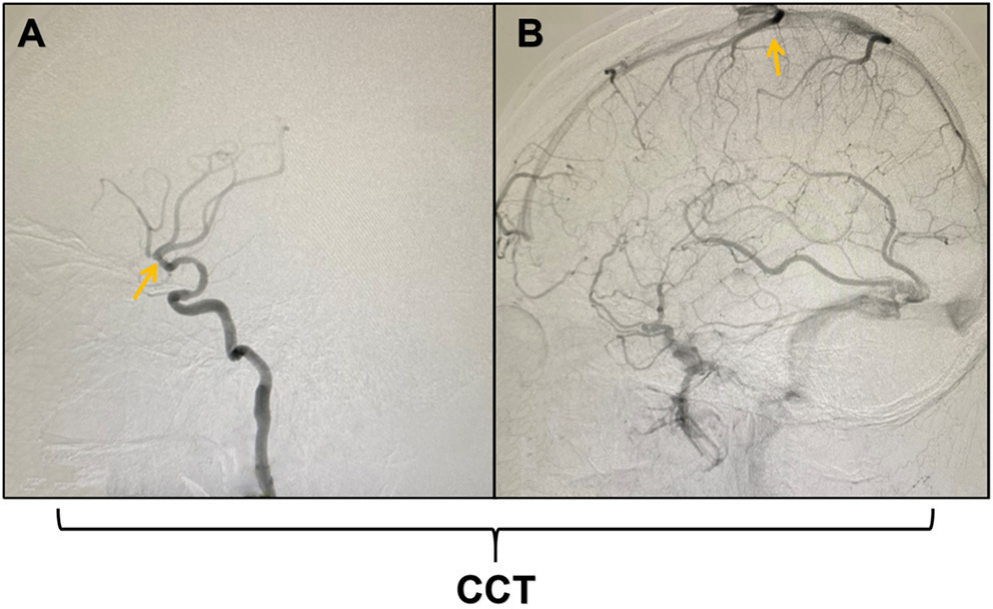
Schematic diagram of the measurement of cerebral circulation time (CCT). **(A)** shows the appearance of the middle cerebral artery (MCA); **(B)** shows the peak intensity of the Trolard vein. The CCT was defined as the time from the appearance of the MCA to the peak intensity of the Trolard vein during digital subtraction angiography (DSA).

During the angiography procedure, the CCT was defined as the time from the appearance of the MCA to the peak intensity of the Trolard vein (Figure 1). In order to reduce the data errors, the CCT of same patient was measured for three times, and then the average of these three measurements was used in the following analysis. The main research indicator of the present study was rCCT (relative CCT), which was calculated with the following formula: rCCT = the CCT of the stenotic side (sCCT)/the CCT of the healthy side (hCCT).

### Treatment Options

All patients received aggressive medical therapy for at least 3 months after the DSA examination. Four of the 5 patients in the DICE (+) group and 27 of the 41 patients in the DICE (–) group received dual antiplatelet therapy (100 mg aspirin and 75 mg clopidogrel per day). The remaining patients all received single antiplatelet therapy. Patients suffering from hypertension, diabetes, or dyslipidemia all received standard drug treatment.

### Statistical Analysis

All statistical analyses were conducted using SPSS 23 for Windows. All continuous variables were tested for normality and expressed as median (interquartile range). Categorical variables were expressed as numbers (%). The differences in demographic and clinical variables between the DICE (–) and DICE (+) groups were analyzed by Mann-Whitney U-tests for continuous variables and Pearson's χ^2^ test or Fisher's exact test for categorical variables. Logistic regression analysis was performed to estimate the odds ratio (OR) for DICE with rCCT and other risk factors as independent variables. *P* < 0.05 was considered statistically significant. Receiver operating characteristic (ROC) curve analysis of rCCT was performed to evaluate the predictive accuracy of DICE in the ND-MCAS patients.

## Results

Between January 2015 and January 2020, 46 patients with ND-MCAS were confirmed by DSA in our hospital, including 30 males (65.2%), with an average age of 55 (48.7, 63) (range 25–87 years), hypertension (45.6%), diabetes (26.1%), dyslipidemia (45.6%) and smoking (26.1%). Eighteen of the patients presented with TIA, and 28 presented with minor ischemic stroke. Among all of the included patients, 5 patients suffered from DICE within 3 months after DSA and were allocated to the DICE (+) group, and the remaining patients without disability were allocated to the DICE (–) group. The demographic and clinical data of the patients is shown in Table 1.

**Table 1:** The demographic and clinical data for patients with and without DICE.

**Characteristics**	**DICE (–) (*N* = 41)**	**DICE (+) (*N* = 5)**	***p*-value**
Age (years)	54 (47.5, 61)	69 (57.5, 77)^*^	0.026
Male	28 (68.3)	2 (40)	0.449
Hypertension	19 (46.3)	2 (40)	1
Diabetes	9 (22.5)	3 (60)	0.197
Dyslipidemia	18 (43.9)	3 (60)	0.836
Cigarette smoking	11 (26.8)	1 (20)	1
**Usage of drugs within 3 months**			
Dual antiplatelet agents	27 (65.9)	4 (80)	0.895
Intensive lipid-lowering	13 (31.7)	2 (40)	1
**Clinical manifestation and score**			
Transient ischemic attack	15 (36.5)	3 (60)	0.598
minor ischemic stroke	26 (63.5)	2 (40)	
NIHSS (baseline)	0 (0, 1)	0 (0, 1)	0.587
NIHSS (3 month)	0 (0, 1)	6 (4.5, 8.5)^***^	<0.001
mRs (baseline)	1 (0, 1)	0 (0, 1)	0.233
mRs (3 month)	1 (0, 1)	3 (2.5, 4)^***^	<0.001
**Image data**			
Stenosis degree (%)	70 (30, 80)	80 (60, 85)	0.391
sCCT (s)	6.83 (6.50, 7.75)	7.67 (6.50, 10.67)	0.258
hCCT (s)	6.33 (5.92, 7.00)	6.25 (4.96, 7.25)	0.480
rCCT	1.08 (1.05, 1.14)	1.30 (1.22, 1.54)^***^	<0.001

* *p < 0.05 vs. DICE (–) group.*

*** *p < 0.001 vs. DICE (–) group.*

*All continuous variables are expressed as median (interquartile range) and categorical variables are expressed as numbers (%). The statistically significant differences between the DICE (–) and DICE (+) groups were assessed by χ^2^-test or Mann-Whitney U-tests. DICE, disabling ischemic cerebrovascular events; NIHSS, National Institutes of National Institutes of Health Stroke Scale; mRs, The Modified Rankin Scale; CCT, cerebral circulation time; sCCT, CCT of stenotic side; hCCT, CCT of healthy side; rCCT, relative CCT = sCCT/hCCT*.

**Figure 2:**
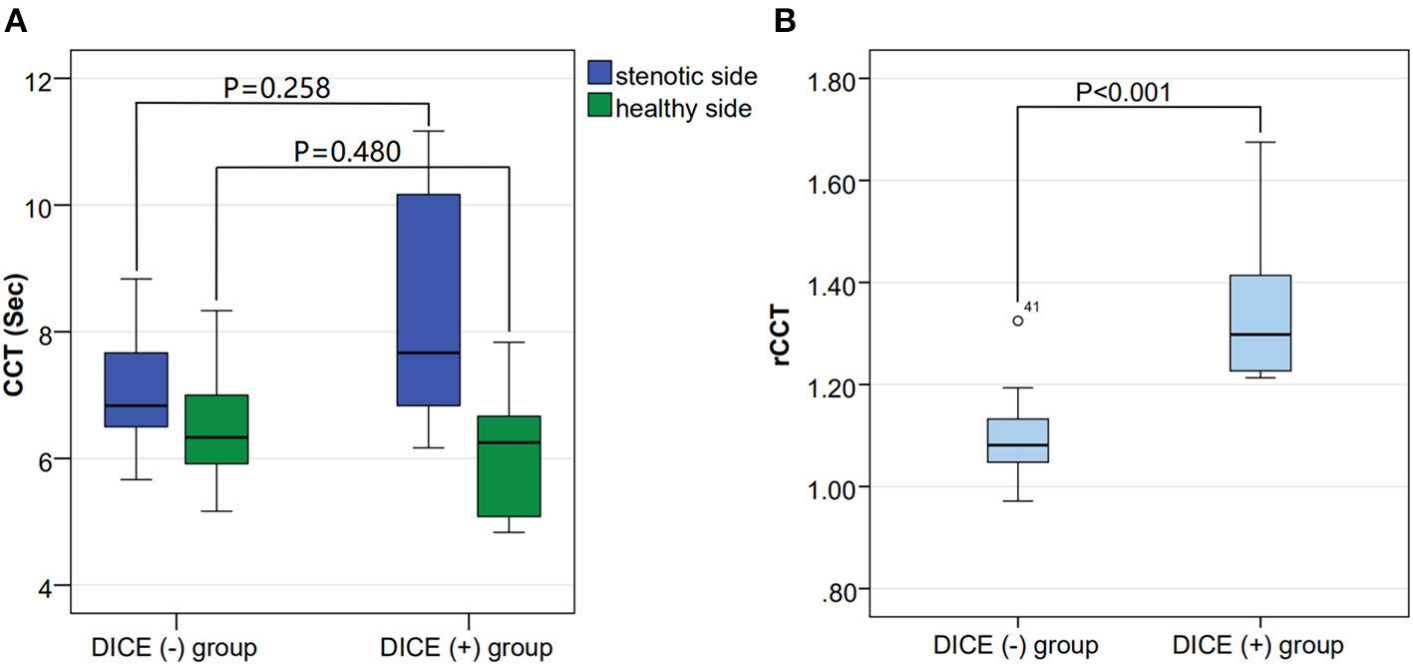
Comparison of the stenotic side CCT (sCCT), the healthy side CCT (hCCT) and the relative CCT (rCCT) among the DICE (–) and DICE (+) groups. **(A)** shows no difference in hCCT [6.33 (5.92, 7.00) vs. 6.25 (4.96, 7.25), *p* = 0.480] and sCCT [6.83 (6.50, 7.75) vs. 7.67 (6.50, 10.67), *p* = 0.258] between the DICE (–) and DICE (+) groups. **(B)** shows a significant difference in rCCT (1.37 ± 0.19 vs. 1.09 ± 0.07, *p* < 0.001) between the DICE (–) and DICE (+) groups.

No significant differences were observed in sex, stroke risk factors, baseline clinical manifestation or usage of drugs between the DICE (–) and DICE (+) groups. The rCCT in the DICE (+) group were prolonged compared with those in the DICE (–) group [1.08 (1.05, 1.14) vs. 1.30 (1.22, 1.54), *p* < 0.001; Table 1, Figure 2]. There was no significant difference in sCCT and hCCT between the two groups [6.83 (6.50, 7.75) vs. 7.67 (6.50, 10.67), *p* = 0.258; 6.33 (5.92, 7.00) vs. 6.25 (4.96, 7.25), *p* = 0.480; Table 1, Figure 2].

**Figure 3:**
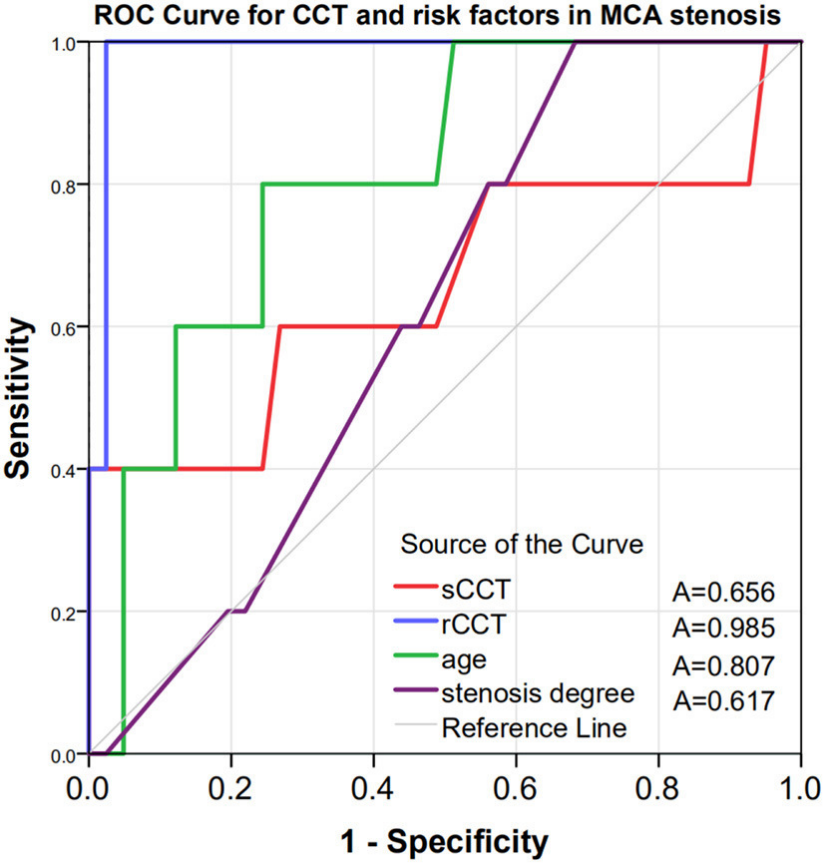
Receiver operating characteristic curves to evaluate the utility of CCT and traditional risk factors in predicting DICE in patients with ND-MCAS. The AUC was 0.985 for rCCT (blue curve), 0.656 for sCCT (red curve), 0.807 for age (green curve) and 0.617 for stenosis degree (purple curve). CCT, cerebral circulation time; sCCT, CCT of the stenotic side; hCCT, CCT of the healthy side; rCCT, relative CCT = CCT of the stenotic side/CCT of the healthy side; A, area under the curve; ROC, receiver operating characteristic; DICE, disabling ischemic cerebrovascular events; ND-MCAS, non-disabling middle cerebral artery stenosis.

Logistic regression analysis showed that after adjustment for possible confounders, namely, age, diabetes, stenosis degree and antithrombotic use, a longer rCCT was independently associated with a higher risk of DICE (OR = 1.273, *p* = 0.019, Table 2). The ROC curves of CCT and the other risk factors for the prediction of DICE are shown in Figure 3. The optimal cutoff point of rCCT (1.20) predicted DICE with 100% sensitivity and 97.6% specificity (Table 3, Figure 3). In contrast, the optimal cutoff point of sCCT (9.5 s) predicted DICE with 40% sensitivity and 100% specificity; the optimal cutoff point of stenosis degree (65%) predicted DICE with 100% sensitivity and 43.9% specificity (Table 3, Figure 3).

**Table 2:** Multivariable logistic regression analysis to evaluate the association between DICE and risk factors, including rCCT.

**Variables**	**Univariate**			**Multivariate model**		
	**OR**	**95% CI**	***p*-value**	**OR**	**95% CI**	***p*-value**
rCCT	1.295	1.053–1.592	0.014	1.273^*^	1.041–1.556	0.019
Age (years) (mean ± SD)	1.093	1.004–1.190	0.040	1.063	0.865–1.306	0.564
Dual antiplatelet therapy	0.482	0.049–4.735	0.531	1.646	0.006–455.233	0.862
Stenosis degree	1.025	0.978–1.075	0.297	1.052	0.857–1.292	0.626
Diabetes	5.333	0.769–36.965	0.090	0.127	0.002–8.309	0.333

* *p < 0.05.*

*DICE, disabling ischemic cerebrovascular events; CCT, cerebral circulation time; rCCT, relative CCT = sCCT/hCCT; OR, odds ratio; CI, confidence interval*.

**Table 3:** ROC curves for traditional risk factors and CCT in the prediction of disabling ischemic cerebrovascular events.

**Variable**	**Traditional risk factors**		**CCT**	
	**Age**	**Stenosis degree**	**sCCT**	**rCCT**
AUC	0.807^*^	0.617	0.656	0.985^***^
Cutoff value	0.556	0.439	0.4	0.976
*p*-value	0.026	0.397	0.259	<0.001
95% CI	0.636–0.979	0.417–0.817	0.338–0.974	0.953–1.000
Sensitivity	80%	100%	40%	100%
Specificity	75.6	43.9%	100%	97.6%

* *p < 0.05*

*** *p < 0.001.*

*ROC, receiver operating characteristic; CCT, cerebral circulation time; rCCT, relative CCT = sCCT/hCCT; AUC, area under the curve; CI, confidence interval*.

## Discussion

Non-disabling ischemic stroke, which indicates a patient is at risk of an early recurrent stroke, can lead to severe disabling events and even death, seriously affecting their quality of life (27–30). Therefore, early identification of such patients is particularly important (31–33). In this study, we provided novel observations by evaluating the usefulness of CCT for the prediction of DICE in ND-MCAS patients.

Compared with other previous studies, we specifically demonstrated the presence of prolonged rCCT in arterial stenosis. Yamamoto et al. detected prolonged CCT measured through visual observation in patients with unilateral occlusive lesions in the ICA or MCA (21). Lin et al. investigated multiple segments of CCT in 25 patients with carotid stenosis and 34 normal control subjects and found that some of the segments of CCT were longer in patients with carotid stenosis than in control subjects (34). In our study, we found that rCCT was a more sensitive marker than sCCT for predicting DICE in ND-MCAS.

Impaired cerebrovascular reserve is the main cause of cerebral infarction. Lin et al. showed that CBF, MTT and Tmax were found to correlate with CCT. Prolonged CCT may reflect the impairment of cerebrovascular reserve (20). This conclusion is in line with our findings, in which 5 of the 46 patients showed significant prolongation of rCCT and subsequently suffered DICE. The rCCT in these 5 patients were significantly increased compared with the patients without DICE. We could conclude that rCCT may reflect the cerebrovascular reserve and predict the occurrence of DICE. At the same time, possibly because rCCT excluded the difference in CCT among individuals, rCCT was found to be more effective than sCCT in predicting DICE (AUC of rCCT = 0.985, *p* < 0.001, AUC of sCCT = 0.656, *p* < 0.259).

Our results also suggested that rCCT (OR = 1.273, *p* = 0.019) was a more sensitive predictor of DICE than the degree of stenosis (OR = 1.025, *p* = 0.626) after adjustment for age, diabetes, usage of dual antiplatelet therapy and stenosis degree. Yong's study showed that prolonged CCT was more closely associated with symptomatic carotid stenosis than stenosis degree or collateral circulation (34). This theory also seems to be applicable in our study for intracranial vascular stenosis.

Scientific statements from the American Heart Association have shown that angioplasty or placement of a Wingspan stent may be warranted for patients with severe stenosis (70–99%) of a major intracranial artery who have progressing symptoms, recurrent TIA or stroke (2). In this study, the risk of DICE occurring in patients with MCA stenosis was assessed in real-time by intraoperative measurement of rCCT. For patients with severe rCCT prolongation, traditional drug therapy may not prevent the occurrence of severe DICE. Thus, with the help of rCCT in the prediction of DICE, the surgeons may prefer immediate intravascular treatment after calculating rCCT, avoiding the additional radiation, contrast agent and economic burden from SPECT, CTP, PWI, and secondary surgery.

The limitations of this study include the small number of cases and the imbalance in the number of cases between the DICE (–) and DICE (+) groups. The patients in the present study were followed up for only 3 months. Longitudinal studies of large cohorts and longer follow-up periods are needed to confirm our results. In addition, collateral circulation may influence the prognosis of the patients (35). The relationship between CCT and collateral circulation was not included in the analysis of this study, and it may need further investigation in the future.

## Conclusions

Prolonged rCCT is an independent positive prognostic factor for the occurrence of DICE in patients with ND-MCAS treated with drugs, and it could be used by surgeons to identify individuals at high risk of DICE during surgery. In patients with a significantly prolonged CCT, angioplasty or placement of a balloon expandable and self-expanding vascular stents may be warranted even when their degree of stenosis is <70%. Future studies with larger sample sizes are required to further verify the sensitivity and accuracy of CCT in the clinical management of stroke.

## Data Availability Statement

The raw data supporting the conclusions of this article will be made available by the authors, without undue reservation.

## Ethics Statement

The studies involving human participants were reviewed and approved by the Ethics Committee of Zhujiang Hospital of Southern Medical University and conducted in accordance with the ethical standards of the 1975 Declaration of Helsinki and the 1999 National Institutes of Health Human Subjects Policies and Guidance. The patients/participants provided their written informed consent to participate in this study.

## Author Contributions

ZC, XW, CL, and QW: conceived and designed the clinical study. ZC, MingL, ZW, MZ, MinzL, GW, SZ, CL, XW, and QW: performed the clinical study. ZC, CL, XW, and QW: analyzed the data. CL, XW, and QW: revised the paper for intellectual content. ZC, CL, XW, and QW: wrote the paper. All authors read and approved the final manuscript.

## Conflict of Interest

The authors declare that the research was conducted in the absence of any commercial or financial relationships that could be construed as a potential conflict of interest.
